# Preliminary Studies into Cutting of a Novel Two Component 3D-Printed Stainless Steel–Polymer Composite Material by Abrasive Water Jet

**DOI:** 10.3390/ma16031170

**Published:** 2023-01-30

**Authors:** Tomasz Szatkiewicz, Andrzej Perec, Aleksandra Radomska-Zalas, Kamil Banaszek, Blazej Balasz

**Affiliations:** 1 Faculty of Mechanical Engineering, Koszalin University of Technology, 75-620 Koszalin, Poland; 2 Faculty of Technology, Jacob of Paradies University, 66-400 Gorzow Wielkopolski, Poland; 3 Doctoral School, Koszalin University of Technology, 75-620 Koszalin, Poland

**Keywords:** abrasive water jet, cutting, metal–polymer composite, modeling, optimization, surface roughness

## Abstract

Composites are materials with a heterogeneous structure, composed of two or more components with different properties. The properties of composites are never the sum or average of the properties of their components. There is a lot of research and many models on the different property assessments of composite materials. Composites are used as construction materials in key areas of technology, including in civil and mechanical engineering, aviation and space technology, and others. This work presents a modern composite material created with 3D-printing technology using the SLM method, and the possibility of its processing with one of the advanced manufacturing technologies, i.e., the Abrasive Water Jet (AWJ). Tests planned using DoE methods were carried out by changing control parameters such as the pressure, abrasive flow, and traverse speed. As a dependent parameter, the surface roughness parameter Sq (squared mean height) was selected and measured in different places of the cut composite. Based on the S/N ratio, the most favorable control parameters of the cutting process were also determined to achieve the lowest roughness of the cut surface. A clear effect of the controlled cutting process on the surface roughness was observed, as well as roughness variation for the metal and polymer component. In addition, the contact surface of the polymer with the metal in the cut zone was analyzed. Analysis of the contact surfaces on the microscope showed that the gap between the polymer–metal contact surfaces does not exceed 2.5 μm.

## 1. Introduction

The development of multilayer composites with the desired characteristics has emerged as a good substitute for expensive materials. Novel advanced engineered materials such as metal–plastic composites are increasingly utilized in the space and aerospace industry. The joining of metal layers flooded in plastic in one hybrid material system ensures higher strength and corrosion properties when compared to their uniform equivalents [1]. The progressive diversity in the use of high-performance materials and the combination of material composites is challenging for ordinary machining methods. Because of the difference in the machining properties of each material phase, conventional treatment often generates damages and faults, causing the increasing cost and decreasing the strength performance of the made part. The conventional techniques for cutting such as the oxy torch or gas/plasma arc yield a poor cut quality and wide heat-affected zones. A real alternative to reduce the cutting issues is Abrasive Water Jet (AWJ) machining [2]. 

AWJ machining is one of the best comprehensive advanced machining processes in several industries for the machining of a variety of engineering materials [3,4,5]. Reaching a maximum depth is a major condition to achieve high efficiency [6,7]. Additionally, AWJ is mostly used for the treatment of hard material [8,9,10] due to its tremendous machining outcomes.

A further advantage of AWJ technology is its environmental friendliness, since amid the known treatment method of machining only plastic processing [11,12] and AWJ cutting [13,14] can stand up to these requirements. 

Among metal-based composites, Metal Matrix Composites (MMCs) are the most popular materials composed of multiple layers of metals with different properties. MMCs produced by the laser processes are increasingly used as additional protective coatings in industry to improve the wear, erosion, and corrosion resistances of components and extend their service life. 

Kovacevic et al. [15] conducted tests of the AISI 420/VC metal matrix composite with a various weight percent (0–40 wt%) of Vanadium Carbide (VC). The influence of carbide content on the microstructure, element distribution, phases, and microhardness were examined in detail. The erosion resistance of the coatings was tested with a high-pressure abrasive water jet (AWJ). The results showed that the erosion resistance of the clad layer was improved with the introduction of the VC. As the VC content increased, the erosion resistance increased. No clear improvement in erosion resistance was observed when the VC fraction was above 30 wt%.

Expanded research into the erosion of the composite of vanadium carbide (VC), titanium carbide (TiC), and tungsten carbide (WC) blended with AISI 420 stainless steel (SS) powders, respectively, to fabricate metal matrix composite (MMC) coatings on the mild steel A36 by using a high-power direct diode laser was presented by Kovacevic et al. [16]. To rate the erosion efficiency, the abrasive waterjet was used for erosion testing conducted at three attack angles, 30°, 45°, and 90°. The results showed that the resistance of 420 SS to erosion was increased at the impingement angles of 30° and 45° after adding the carbides. At the 90° attack angle, the addition of VC and TiC increased the erosion resistance of the 420 SS. For all layers, the VC-reinforced coating demonstrated the best anti-erosive efficiency at all attack angles.

Ishfaq et al. [17] tested the influence of important control parameters of abrasive waterjet machining (AWJM) to measure their impact during the machining of stainless-clad steel utilizing the Taguchi design. Tests results were processed using statistical and microscopical evidence. The optimal combination of control parameters resulting in the minimum magnitude of depth at both (the stainless steel layer and mild steel layer) the clad layers was developed and experimentally confirmed. The magnitude of the cutting depth realized at the stainless steel layer (S-SL) and mild steel layer (M-SL) was significantly reduced to 5 μm and 4 μm correspondingly, at the optimal control parameter combination.

As the AWJM is characterized by several control parameters, selecting the best set of these to achieve the maximum depth of cut is an arduous and time-consuming process. Thus, Mohankumar et al. [18] chose process variables through suitable modeling techniques. They used a semi-empirical model using Buckingham’s pi theorem to predict the depth of cut during the AWJM of reinforced aluminum metal matrix composites. Experiments were designed according to the Box–Behnken method, and cutting tests were performed on unreinforced aluminum and a 5%, 10%, and 15% volume fraction of boron carbide (B_4_C)-reinforced 6063 aluminum alloys. The experimental results of the depth of cut at different levels of the process parameters were analyzed with ANOVA. Based on the experimental results, the combinations resulted in a higher depth of cut. The models’ predictions were in good agreement with the experimental data under the corresponding conditions.

Srivastava et al. [19] published the effects of cutting the previously developed sample of the hybrid metal matrix composite A359/B_4_C/Al_2_O_3_ by the abrasive waterjet process. The different samples with changed proportions of reinforcement from 2% to 4% were used in this study. The result reveals that rough cutting with the average surface roughness value ranges from 7 to 9 μm for the selected samples.

Hashish et al. [20] presented the machinability study of thermoplastic Titanium Graphite (TiGr) FML. The machinability of the outline thickness (7.6–10.5 mm) of TiGr through the Abrasive Waterjet (AWJ) process was studied in conditions of machined kerf characteristics and taper ratio and surface roughness. The effects of a wide range of process parameters were investigated such as the effect of the geometric and kinetic variables including the abrasive load ratio on the treatment quality. Predictive mathematical regression models were developed through Analysis of Variance (ANOVA) to optimize the process. A comparison between conventional machining and AWJ treatment of TiGr showed the predominance and higher efficiency of AWJ with less damage.

The study of the cutting efficiency of the stacked Ti6Al4V and CFRP system using the abrasive water jet (AWJ) technology was presented by Pahuja and Ramulu [21]. They conducted experiments with AWJ cutting materials in the form of a titanium pile and monolithic CFRP. A strong linear relationship was identified between the flux power-to-velocity ratio and the cut depth for CFRP and titanium individually, which was then used to predict the depth of cut in the stack Ti-CFRP configuration.

Naveen Reddy et al. [22] conducted research and optimization of AWJ processing parameters on a glass–epoxy reinforced aluminum composite of the GLARE type. It was subjected to a drilling operation with various control (input) parameters such as the nozzle–material distance, feed, abrasive mass flow rate, and water pressure, and with output parameters such as delamination and surface roughness.

Gnanavelbabu et al. [23] presented a study of cutting the hybrid composites AA6061-B4C-hBN. They determined the effects of the process parameters such as the size, abrasive flow rate, pressure, and feed in an AWJ machining process. They also showed that pressure and feed were the most important parameters that influenced the effects such as the surface angle of the cut groove and the roughness of the surfaces.

Putz et al. [24] published the high-accuracy machinability results for 2D- and 3D-layer composites out of CFRP/light metals such as aluminum or magnesium by the AWJ. Different cutting strategies were established as well as the impact of the process on the joining area and changed properties of the machined materials.

Singh et al. [25] presented the study of AWJ cutting of single- and double-layered structures with two varied materials such as aluminum, steel, and rubber. Authors conducted analyses of the geometry of the slot generated in the multilayer structures and analyses of a stack of multiple materials with each material having different mechanical properties. The research was also covering an analysis of the role of an interfacial adhesive layer on kerf profile properties, and the preferential orientation of multi-layered structures and stacked materials, to AWJs for producing a near uniform cut slot profile.

In addition, Vedernikov et al. [26] investigated the relationships between the pulling speed, morphology, and mechanical properties of pultruded glass fiber/vinyl ester structural composites. They showed that increasing the pulling speed significantly affected the morphology and mechanical properties of the composites. The size, number, and density of bubbles, blisters, and voids increased with an increase in the pulling speed from 200 mm/min to 600 mm/min, and further to 1000 mm/min. Additionally, they also demonstrated that high-speed pultrusion makes it possible to increase the pultrusion output by at least 1.7 times without compromising the mechanical performance of the produced profiles as compared to their regular speed-produced counterparts.

However, Minchenkov et al. [27] examined the properties of pultruded thermoplastic glass fiber/polypropylene flat laminates based on two types of pre-consolidated tapes produced by two different manufacturing processes. They investigated the cross-sectional morphology and surface roughness of both pultruded flat laminates and source pre-consolidated tapes. They also conducted mechanical tests on pultruded laminate specimens under compression, tension, and flexure loadings. The results showed that defects present in pre-consolidated tapes impair the mechanical characteristics of the final composites.

Due to the outstanding mechanical properties of TPMS structures and honeycomb patterns, attempts have been made also to use them as 3D-printed formwork and reinforcement in concrete beam application. Katzer et al. [28,29] and Stokratko et al. [30,31] examined the mechanical properties and fatigue/damage models of composites of 3D-printed frames (ABS) and concrete. The results showed that 3D-printed frames, due to their complex shape, can successfully replace steel reinforcements in civil engineering applications.

On state-of-art analysis, the AWJ appears applicable for machining composites out of polymers and metals, even though their properties can be extremely varying. AWJ can be preferred for the treatment of multi-layered structures. However, the randomly deter-mined nature of the abrasive water jet (AWJ) interacting on a multi-layered structure (MLS) can generate some problems with non-regular cut slot geometry. 

Furthermore, AWJ shows useful process effects due to its minimal mechanical and thermal impact during treatments compared to ordinary cutting processes. Despite that AWJ machining guarantees abundant benefits for the treatment of a polymer–metal composite, some potential problems need to be solved. On the one hand, the machining of different materials could be the reason for the differences in roughness and kerf geometry on the cut surface of each constituent component. On the other hand, water and the abrasiveness may impair the contact zone between the two components. Both phenomena could have an impact on the deterioration in the product quality and increase the machining time.

Therefore, it was decided to conduct AWJ cutting tests on a composite consisting of elements with different physicochemical properties and an unfolded 3D structure. As a result of the differences in the mechanical properties of the constituent materials of the composite, a special method of processing (cutting) is required. Conventional material separation processes do not provide an adequate quality and performance. 

In addition, the machinability by the AWJ is a valuable property in the further operation of components, especially a large-sized one made of such composite material. When a large component is damaged, it will be possible to cut out the damage and insert a repair patch, without having to replace the entire large-sized component. This study tested the influence of the most important control parameters of the cutting process on the quality of the cut-side surface of the composite. The focus was on determining the behavior of the material at the metal–polymer interfaces.

## 2. Materials and Methods

### 2.1. Cut Material

**Figure 1:**
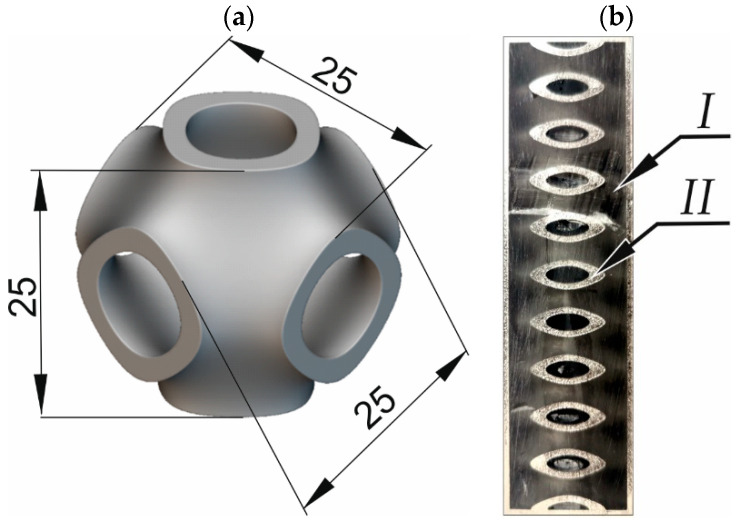
Sample view of: (**a**) an individual Schwarz-P cell, (**b**) a real 3D metal composite: I—polymer, II—3D-printed stainless steel 316L.

The cut material is a metal–polymer composite. The metal framework is in the shape of periodically repeating Schwarz-P cells (Figure 1a), surrounded by outer walls acting as a mold (Figure 1b) for the epoxy polymer filling. The Schwarz-P elementary cell is described by Equation (1) and belongs to the TPMS surface class [32].

(1) $$t^{2}={\left(\cos\left(x\right)+\cos\left(y\right)+\cos\left(z\right)\right)}^{2}$$

The value of the parameter *t* in Equation (1) controls the thickness of the cell wall, and in this experiment, it is set so that the wall of the metallic framework is 2 mm thick. The CAD model of the specimen was generated using nTopology software [33] as a .stl file. The samples were fabricated from stainless steel 316L powder using an ORLAS CREATOR^®^ (O. R. Lasertechnologie GmbH, Dieburg, Germany) selective laser melting (SLM) machine [34]. 

The printed mold was then flooded with *Epidian 5* polymer resin from Ciech Sarzyna/Poland. This polymer is characterized by a high hardness and good adhesion to metals. The resulting composite consisted of two materials with different strength properties and densities (7.98 kg/dm^3^ for 316L stainless steel and 1.15 kg/dm^3^ for the polymer). The shape of the metallic skeleton determined the high mechanical properties of the composite, while filling with the relatively brittle Epidian 5 polymer significantly increased the mechanical properties of the composite with a slight increase in its relative density. It becomes a significant challenge to develop cutting technology for such composites, and with such diverse constituent materials, waterjet technology is an interesting alternative to conventional cutting methods. 

### 2.2. Abrasive Material

In tests, the almandine garnet type J80A from Jiangsu deposit, China was utilized. Almandine is the iron alumina garnet. Its chemical formula is Fe_3_Al_2_(SiO_4_)_3_. Deep red colored inclining to purple crystal forms are well-formed dodecahedral and trapezohedron shaped (Figure 1a), and occasionally are modified combinations of the two [35]. Crystals may be striated or have stepped growth layers and are sometimes warped into rounded ball-like forms. 

The close to isometric shape of the almandine grain and its relatively high hardness and density often make it used as an abrasive agent in abrasive jet machining.

**Figure 2:**
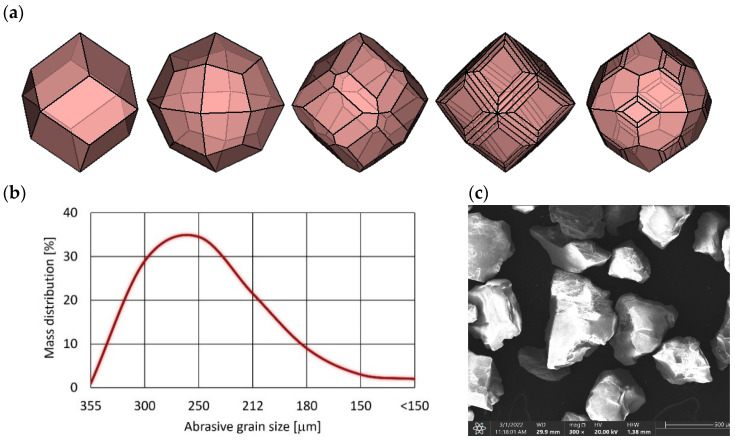
The J80A garnet abrasive: (**a**) the crystal shapes, (**b**) the grain size distribution, (**c**) the sample view of the grains.

Almandine garnet type J80A is a pure natural mining abrasive. Its chemical composition (Table 1) and particle distribution (Figure 2b) can vary slight within a certain range, but the performance is stable.

### 2.3. Research Method

**Figure 3:**
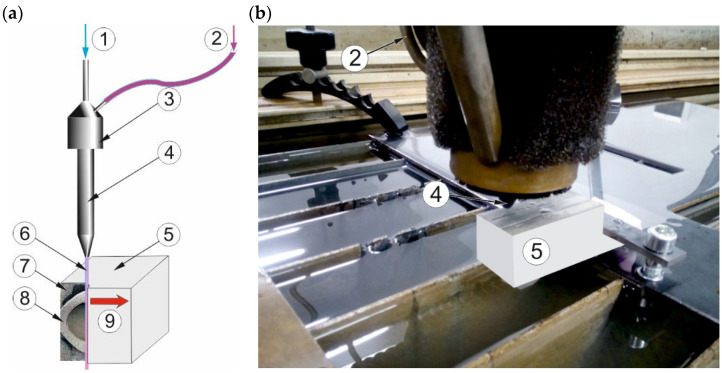
The test rig: (**a**) the schematic, (**b**) the real view; 1. High-pressure water inlet, 2. Abrasive inlet, 3. Mixing chamber, 4. Focusing tube, 5. Cutting material, 6. Abrasive Water Jet, 7. Polymer, 8. Stainless steel, 9. Traverse speed direction.

The tests were executed on the Omax Jet Machining Center 60120. Material samples were cut by the jet formed in focusing the tube and directing it perpendicular to the top surface of the workpiece, causing relative linear moving (Figure 3).

**Figure 4:**
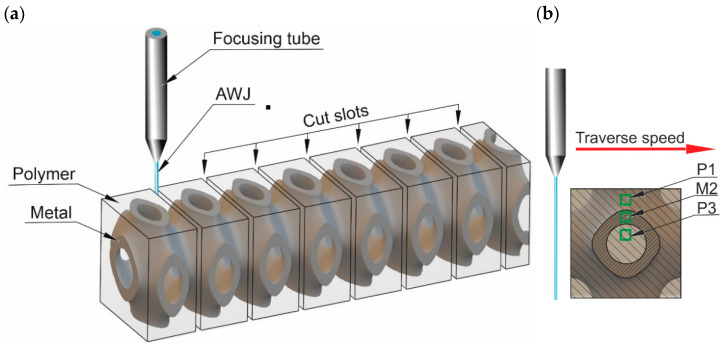
Details of the AWJ cutting tests: (**a**) the cut slot locations, (**b**) the roughness measuring area locations.

Since the structure of the composite subjected to the study is variable in 3D space as perfectly shown in Figure 4a, for cutting, the recurring areas in the structure were selected. This allowed to test analogous properties of the composite in the cross-sectional plane.

To evaluate the surface roughness of the cut slot, three areas (Figure 4b) were selected:

- P1—the polymer area located in the middle of the distance from the top sample surface to the metal part,
- M2—the area in the center of the cut metal part,
- P3—the polymer area located in the middle of the sample.

**Figure 5:**
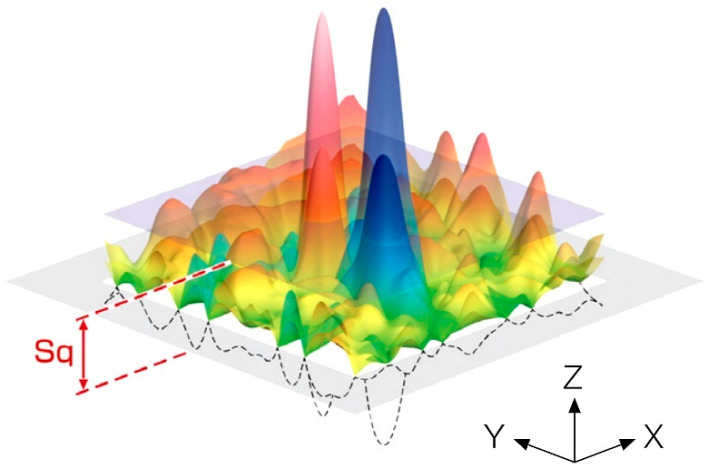
Graphical illustration of the surface roughness parameter *Sq* [39].

To compare the test results, the state of the cutting surface and especially the roughness of the kerf surface [37,38] were evaluated using the *Sq* parameter. Commonly, abrasion progresses from the surface’s highest position. The use of height distribution-based factors is effective in the estimate of the abrasion status. *Sq* exemplifies the root mean square value of ordinate values within the designation area (Figure 5). It is the 3D counterpart to the standard deviation of heights of the profile roughness and is an easy-to-handle statistical parameter. As the factor *Sq* uses maximal and minimal height values, the constancy of the results may be harmfully influenced by measurement noise. 

This parameter is established solely by the distribution of the height data. Therefore, the characteristics of horizontal features are not reflected in this parameter. The *Sq* index was calculated based on Equation (2).

(2) $$Sq=\sqrt{\frac{1}{A}\iint_{A}^{}Z^{2}\left(z,y\right)dxdy}$$

While the height distribution is normal, the relation of *Sq* to the basic roughness parameter *Sa* becomes *Sq* = 1.25·*Sa*. Roughness measurements were made using a non-contact method on an Olympus DSX1000 digital optical microscope. 

The design of the experiment (DOE) method was used to limit the number of the tests and to shorten their time. The cutting research was carried out according to the L9 orthogonal array. This design model covered three of the control parameters: pressure, traverse speed, and abrasive expenditure, which each took 3 levels. Control parameter ranges were chosen based on prior papers [40,41] and the works of different researcher teams: Hocheng et al., [42], Hlavacova et al. [43], and Spadlo et al., [44]. The cutting process was carried out with the following range parameters:

- Traverse speed: 100–300 mm/min,
- The abrasive expenditure: 250–450 g/min,
- Pressure: 360–400 MPa.

Obviously, the factor *Sq* was used as an output parameter and the roughness was measured in areas according to Figure 4.

## 3. Results and Discussion

The results of the tests with the control parameters are given in Table 2. The Delta statistic was used for determining the impact ranking of individual control parameters and was calculated as the highest minus the lowest average for each factor. Assignation ranks were based on Delta values. So, rank = 1 Delta achieves the highest value, and if rank = 2 then Delta is the second highest, and so on. Use the level averages in the response tables to determine which level of each factor provides the best result [45]. 

For all three measurement areas, the influence of the control parameters was analogous. Traverse speed was the most significant factor influencing roughness. Meanwhile, pressure respectively has a sub-significant effect on roughness. Abrasive concentration has the least significant influence on roughness. Details of the control parameters’ rank are presented in Table 3.

**Figure 6:**
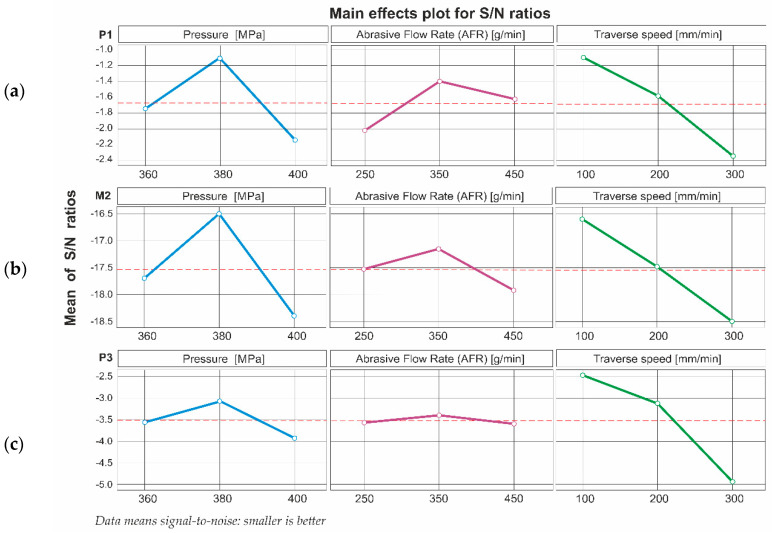
The influence of control parameters on the S/N ratio for surface roughness: (**a**) P1 area, (**b**) M2 area, (**c**) P3 area.

A graphical illustration of the dependence of the S/N ratio on process control parameters is shown in Figure 6. Graphs confirm similarity in the influence of control parameters in each tested area.

For all three measurement areas, the highest S/N ratio values were achieved for the following parameters:

- Pressure: 380 MPa,
- abrasive output: 350 g/min,
- traverse speed: 100 mm/min.

It is for this combination of control parameters that optimum machining results are expected to be achieved. 

Under these conditions, a prediction of the output parameter values was made. The next step was using the control parameter values determined in this way, another cutting test was carried out and the surface roughness was measured in analogous areas. 

**Figure 7:**
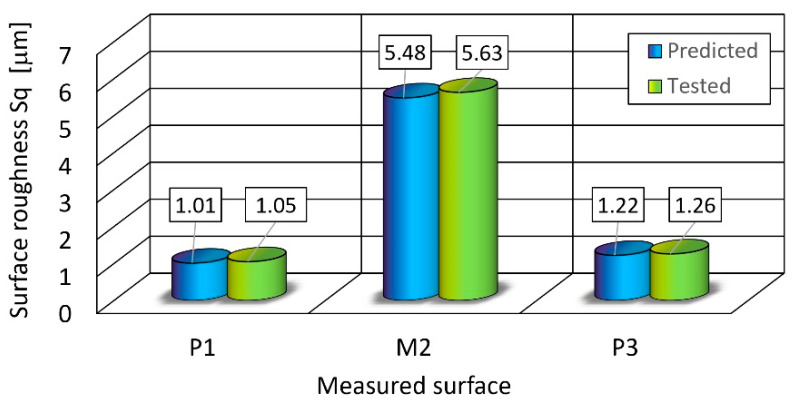
The predicted and tested surface roughness.

A comparison of the values obtained is shown in Figure 7. The differences in the predicted values and those achieved in the control test do not exceed 4%.

**Figure 8:**
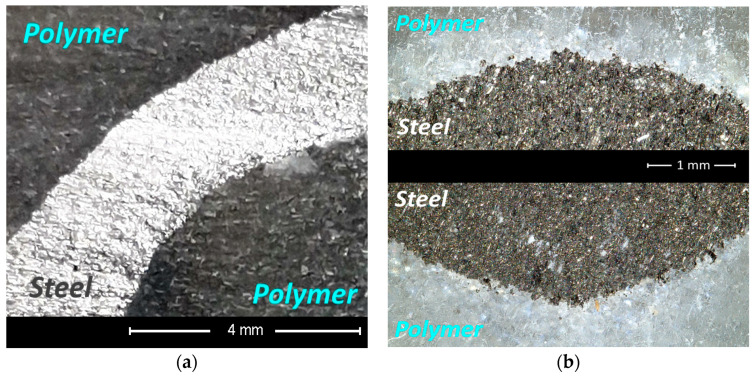

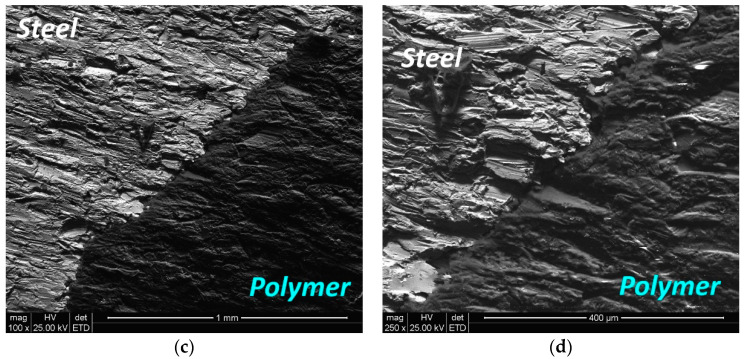
A sample of cut surface: (**a**) the optical microscope view, (**b**) the detailed optical microscope view ×80, (**c**) the SEM microscope view ×100, and (**d**) the SEM microscope view ×250.

An example view of the surface cut under optimal parameters is shown in Figure 8. The characteristic erosion marks left by the abrasive grains on both the polymer and metal surfaces can be seen here.

**Figure 9:**
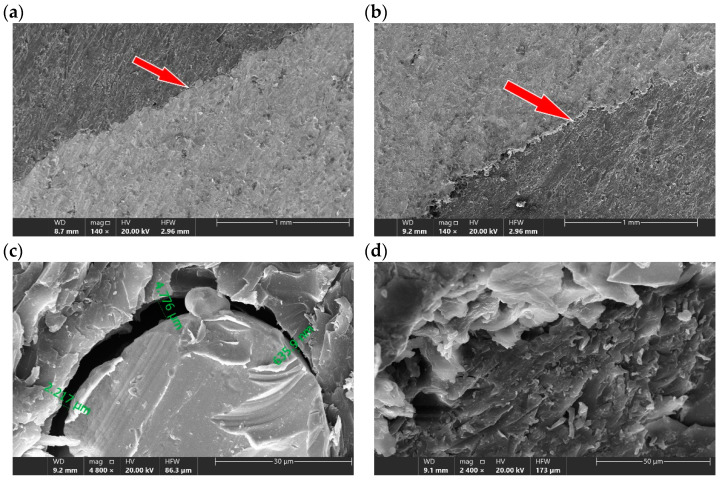
An exemplary view of the raw surface after cutting at the: (**a**) polymer–metal interface, (**b**) metal–polymer interface, (**c**) detail of the polymer–metal interface and the observed gap, and (**d**) detail of the metal–polymer interface.

Particularly noteworthy is the polymer–metal contact zone, because here it is expected to see the effect of the separation of the composite by the penetrating action of the water jet. Observations of the contact surfaces on the SEM microscope on the raw surface, directly after cutting showed (Figure 9) that the gap between the polymer–metal contact surfaces does not exceed 5 μm (Figure 9c). Red arrows indicate the observation area under high magnification. In the case of metal–polymer surface contact, no gap was observed (Figure 9d)

**Figure 10:**
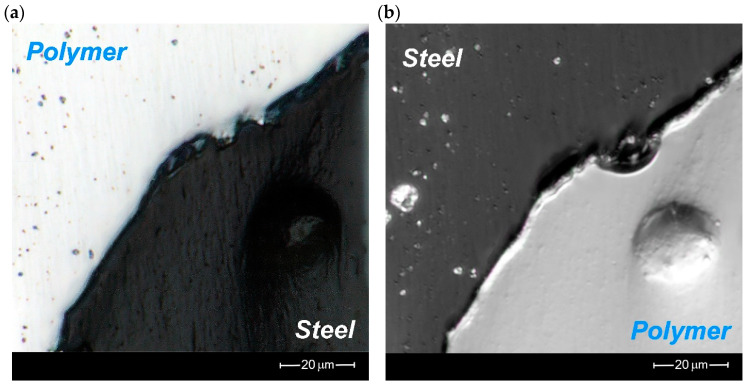
A detailed view of the observed gaps on the prepared surface by polishing after cutting at the (**a**) polymer–metal interface and (**b**) metal–polymer interface.

Figure 10 shows details of the metal–polymer interface after metallographic grinding to eliminate the possibility of shading of the contact area by plastic deformation of the metal. Example measurements of the gap sizes, showing in Table 3, confirm previous observations made on the raw surface directly after cutting. 

The obtained gap at the polymer–metal interface is almost 2.5 times larger than in the case of the metal–polymer interface (Table 4). The observed phenomenon is mainly related to the different erosion resistances of the individual composite components. At the start of the cutting process, the AWJ erodes an easily machinable polymer, and next hits a more resistant component—stainless steel. It begins to penetrate the more easily eroded area of the polymer–metal interface. Hence, a larger gap is created. In the case of further eroding, after cutting through the stainless steel component, the AWJ encounters the easily machinable polymer again and is not forced to penetrate the metal–polymer interface.

## 4. Conclusions

The test results demonstrate the appropriateness of the AWJ for cutting 3D metal–polymer composites.

It was proven that both the influence of the abrasive particles and the water were not harmful effects on the contact area of components in this material. The change in the surface roughness of both components of the 3D composite under tested control parameters is also similar.

The minimal values of the surface roughness were achieved for the following parameters:

- pressure: 380 MPa,
- abrasive output: 350 g/min,
- traverse speed: 100 mm/min.

Under these conditions, surface roughness *Sq* for the metal components achieved a level of 5.6 μm and *Sq* for the polymer components did not exceed a 1.26 μm level. 

In contrast, the differences in predicted and measured values for the optimal control parameters did not exceed 4% for the *Sq* factor.

The microscope analysis of the contact surfaces showed that the average gap between the polymer–metal contact surfaces did not exceed 2.35 μm and between the metal–polymer it was equal to 0.71 μm.

Further research with more differentiated geometries of 3D composites, other output parameters such as the kerf width, and the efficiency factor with control parameter optimization will be required to generalize and model the process, because existing effects are very promising for machining these materials with the AWJ. The effect on composite strength of differences in gap sizes between the polymer–metal and metal–polymer interfaces will also be the subject of further research.

## Figures and Tables

**Table 1:** Properties of J80A garnet [36].

Mineral Composition [%]						
Amandine	Ilmenite	Omphacite	Rutile	Quartz	Hornblende	Free Silica
90–96	1.0	1.5	0.6	<0.1	<0.5	<0.5%
**Chemical Composition [%]**						
Fe_2_O_3_	SiO_2_	TiO_2_	Al_2_O_3_	FeO	CaO	MgO
17	39	0.05	21	8	9.5	5
**Physical Characteristics**						
Density [kg/dm^3^]	Bulk Gravity [kg/dm^3^]	Mohs Hardness	Color	Grain shape	Conductivity	Acid solubility (HCL)
3.8–4.1	2.3–2.4	7.5–8.0	Dark red	Sub angular	<25 S/m	<1.0%

**Table 2:** Results of tests with the control parameter data.

No	Pressure [MPa]	AFR [g/min]	Traverse Speed [mm/min]	*Sq* P1 [μm]	*Sq* M2 [μm]	*Sq* P3 [μm]
1	360	250	100	1.18	6.88	1.31
2	360	350	200	1.21	7.32	1.44
3	360	450	300	1.28	8.94	1.81
4	380	250	200	1.14	6.63	1.39
5	380	350	300	1.18	7.16	1.61
6	380	450	100	1.09	6.29	1.29
7	400	250	300	1.49	9.29	1.88
8	400	350	100	1.13	7.12	1.39
9	400	450	200	1.25	8.64	1.48

**Table 3:** Response Table for Signal to Noise (S/N) Ratios.

	P1			M2			P3		
Level	Pressure	AFR	Traverse Speed	Pressure	AFR	Traverse Speed	Pressure	AFR	Traverse Speed
1	−1.746	−2.013	−1.083	−17.69	−17.51	−16.59	1.520	1.527	1.330
2	−1.108	−1.385	−1.577	−16.50	−17.15	−17.48	1.430	1.480	1.437
3	−2.154	−1.610	−2.349	−18.38	−17.91	−18.50	1.583	1.527	1.767
Delta	1.046	0.628	1.266	1.88	0.76	1.90	0.153	0.047	0.437
Rank	2	3	1	2	3	1	2	3	1

**Table 4:** Details of the gaps between the metal and polymer.

Test	Unit	1	2	3	4	5	6	7	8	9	10	Ave.
Polymer/Steel	[μm]	2.31	3.56	2.09	1.60	1.68	1.91	2.52	2.94	2.46	1.75	2.35
Steel/Polymer	[μm]	0.55	0.61	0.79	0.97	0.84	0.58	0.56	0.7	0.82	0.64	0.71

## Data Availability

Not applicable.
